# Retrospective cohort study on preparation regimens for frozen embryo transfer

**DOI:** 10.1530/RAF-21-0044

**Published:** 2021-11-23

**Authors:** Monique Atkinson, Jenny Crittenden, Howard Smith, Cecilia Sjoblom

**Affiliations:** 1 Westmead Fertility Centre, Westmead Reproduction and Perinatal Centre, Faculty of Medicine and Health, University of Sydney, Westmead Hospital, Corner of Hawkesbury and Darcy Roads, Westmead, New South Wales, Australia

**Keywords:** letrozole, frozen embryo transfer, endometrial preparation, live birth rate

## Abstract

The objective of this study was to examine the pregnancy outcomes from frozen embryo transfer (FET) cycles using different endometrial preparation regimens, compared to ovulation induction with letrozole (letrozole OI). A retrospective cohort study was conducted at a fertility centre in Sydney, Australia, on 6060 FET cycles. The cycles were stratified into one of four ways to achieve endometrial preparation. These were either a natural, letrozole OI, OI with follicle-stimulating hormone (FSH OI) or a programmed cycle. The primary outcome was live birth rate (LBR) per embryo transfer. Secondary outcomes included clinical pregnancy and biochemical pregnancy rates, adverse events including miscarriage, ectopic pregnancy, stillbirth, neonatal death and multiple births. Ovarian stimulation parameters were also analysed including the time taken to reach the luteal phase and the number of blood or urine tests required for monitoring the cycle. The results of the study showed that the LBR following letrozole OI cycles was higher when compared to natural cycles (odds ratio (OR): 1.27 (1.07–1.49)) and programmed cycles (OR: 2.36 (1.67–3.34)). There was no significant difference between letrozole OI and FSH OI LBR (OR: 0.99 (0.76–1.28)). An improved LBR with letrozole OI compared to natural cycles was maintained when only women with a normal length cycle were considered (OR: 1.44 (1.10–1.89)). There was a significant reduction in miscarriage rates when letrozole OI was compared to programmed cycles (OR: 0.46 (0.26–0.83)). It was therefore concluded that the use of letrozole OI for endometrial preparation in an FET cycle may be associated with higher LBR and lower miscarriage rate, compared to using a programmed cycle.

**Lay summary:**

Couples suffering from infertility frequently try to conceive following the transfer of an embryo which has been frozen during an *in vitro* fertilisation cycle. Embryos will only lead to a pregnancy if the woman’s womb lining has particular characteristics that allow it to accept the embryo. Despite the thousands of frozen embryo transfer cycles carried out across the world, it is not known how best to prepare a woman’s lining so it has these particular characteristics. This study looked at the pregnancy outcomes of 6060 cycles to compare four different ways to prepare a woman’s womb lining. These included relying on a woman’s natural menstrual cycle, or using an oral medication called letrozole, or injectable medicine called follicle-stimulating hormone, or oestrogen and progesterone hormonal medications. The comparison found that using letrozole before transfer of a frozen embryo may be associated with higher rates of a live birth for some women.

## Introduction

Optimal preparation of the endometrium and its synchronisation with the embryo to be transferred is imperative to assure the success of frozen embryo transfers (FETs). There is a paucity of well-designed studies which aim to answer the question of which approach to endometrial preparation achieves the best pregnancy outcomes following FET (Groenewoud *et al.* 2013, Ghobara *et al.* 2017, Glujovsky *et al.* 2020). Common regimens include using a natural cycle with or without human chorionic gonadotrophin (hCG) trigger or a programmed cycle using hormone therapy with or without gonadotrophin-releasing hormone (GnRH) agonist suppression (Ghobara *et al.* 2017). Alternatively, follicle stimulating hormone (FSH) may be used to induce ovulation. Letrozole is an aromatase inhibitor which has been used for ovulation induction since the early 2000s (Casper & Mitwally 2011). Its use however for endometrial preparation in FET cycles is less well described yet has shown promising results (Table 1).

**Table 1:** Studies published regarding using letrozole OI for endometrial preparation prior to FET. The complete citations are listed in the references list. The study published by Li *et al.* in 2014 is represented in two parts (part A and part B) as there were two study designs embedded in one publication.

Reference	Study protocol			Regimens compared to letrozole	Significant results	Live birth rate
	Design	Subjects, *n*	Description			
Hosseini-Najarkolaei *et al.* (2020)	RCT	120	Women with PCOS	Programmed	No significant difference	Not reported
Samsami *et al.* (2019)	RCT	162	Women with regular cycles	Programmed	No significant difference	Not reported
Zhang *et al.* (2019)	RC	2664	Women with PCOS	Programmed	Less miscarriages with letrozole	Improved with letrozole (once adjusted for confounding factors)
Tatsumi *et al.* (2017)	RC	110,722 cycles		Natural Programmed	Improved pregnancy outcomes with letrozole: CP, CP with FHB, MC	Improved with letrozole
Aleyasin *et al.* (2017)	RCT	100 cycles		Programmed following GnRH agonist downregulation	Worse implantation rate with letrozole	No significant difference
Sibai *et al.* (2016)	RC	197 cycles		Programmed	Improved OPR with letrozole	Not reported
Li *et al.* (2014)	RCT	713	Women with ovulation disorders	Programmed	Improved pregnancy outcomes with letrozole: Implantation rate, CP, MC	Improved with letrozole
Li *et al.* (2014)	PC	876	Normo-ovulatory women	Natural cycle	No significant difference	No significant difference
Hu *et al.* (2014)	RC	120	Women with PCOS	Programmed, hMG stimulation	Improved pregnancy outcomes with letrozole: CP, OPR	Not reported
Patel *et al.* (2011)	RC	17	Women with endometriosis	‘Standard hormone replacement’	Improved CPR with letrozole: CPR	Not reported
		105	Women without endometriosis			

CP, clinical pregnancy; CP with FHB, clinical pregnancy with fetal heart beat; OPR, ongoing pregnancy rate; MC, miscarriage; LBR, live birth rate; RCT, randomised controlled trial; RC, retrospective cohort; PC, prospective cohort; PCOS, polycystic ovarian syndrome.

Histological and molecular studies have demonstrated that different types of controlled ovarian stimulation can affect the timing and quality of endometrial preparation (Simon *et al.* 1995, Wu *et al.* 2020). Hence, the type of endometrial preparation that is used and its resulting hormonal profile has the potential to affect the success rates of embryo transfer.

Letrozole acts by preventing the conversion of androgens to oestrogens in ovarian follicles, peripheral tissues and the brain. This results in a lower level of circulating oestrogens and a higher level of intra-ovarian androgens. The lower oestrogen level releases the hypothalamic–pituitary axis from negative feedback such that a surge in FSH occurs. The FSH surge leads to mono-follicular growth and ovulation.

A recent Cochrane review on endometrial preparation in FET cycles concluded that it was uncertain if letrozole OI improved the live birth rate (LBR) (Glujovsky *et al.* 2020). The review included one randomised controlled trial (RCT) only, comparing LBR in letrozole OI cycles to programmed cycles (Aleyasin *et al.* 2017). Other RCTs not included in the Cochrane review have reported conflicting outcomes. Li and co-workers demonstrated a significantly improved LBR with letrozole OI compared to programmed cycles (Li *et al.* 2014). More recently published RCTs in women with regular cycles (Samsami *et al.* 2019) or in women with polycystic ovarian syndrome (Hosseini-Najarkolaei *et al.* 2020), however, did not identify any significant difference in pregnancy outcomes when comparing letrozole OI to programmed cycles (Table 1).

To date, there have also been a number of retrospective and prospective cohort studies examining letrozole as an agent for endometrial preparation prior to FET. In general, there has been a consensus that the use of letrozole OI leads to improved FET cycle outcomes (Patel *et al.* 2011, Hu *et al.* 2014, Li *et al.* 2014, Sibai *et al.* 2016, Tatsumi *et al.* 2017, Zhang *et al.* 2019) (Table 1).

The current study aimed to describe the pregnancy outcomes and ovarian stimulation parameters of using letrozole OI as a means of endometrial preparation by retrospectively analysing the cohort data at our institution. It further aimed to examine some of the parameters of the cycles, including the time required to reach the commencement of the luteal phase, in order to guide the selection of women who are appropriate to proceed to embryo transfer.

## Materials and methods

### Study design and population

Women undergoing FET between 2013 and 2018 at Westmead Fertility Centre, Sydney, Australia, were included in this retrospective cohort study. Data were collected and analysed regarding women’s demographics, aetiology for infertility, treatment cycle details and pregnancy outcomes.

Pregnancy outcomes were defined as per the European Society on Human Reproduction and Embryology (ESHRE) glossary consensus (Zegers-Hochschild *et al.* 2017).

Ethical approval for the study was obtained from Western Sydney Local Health District Human Research Ethics Committee (QA 5589).

### Frozen embryo transfer cycle regimens

The regularity of a woman’s natural cycle guided the choice of protocol used to prepare women’s endometrium prior to FET. The natural cycle was used in women with regular cycles, while letrozole was prescribed to women with irregular or lengthy cycles. FSH OI was used in women who did not respond to the letrozole approach in the previous cycle. Programmed cycles were used in amenorrheic or peri/postmenopausal women. The choice of protocol for some women differed from these general principles as ultimately the choice of protocol used was left up to the treating clinician.

### Letrozole OI and natural cycle protocols

If prescribed letrozole, women took either 2.5 or 5 mg letrozole daily on day 2–6 of their cycle, and tracking was commenced from day 9 to 10. In a natural cycle, tracking began 16 days prior to their expected next menstrual period based on their previously observed cycle length. Tracking was performed by monitoring oestradiol (E2), luteinising hormone (LH) and progesterone (P4). Once E2 levels were sufficient (>400 pmol/L), the LH peak was identified using urinary samples.

### FSH OI protocol

Women commenced FSH (Gonal-F, Merck, Australia or Puregon, Merck Sharp & Dohme (MSD), Australia) on day 2–3 of their cycle at a dose decided by the treating clinician, most commonly 50–100 IU, guided by markers of the ovarian reserve. Tracking started on day 5–7 of stimulation and was performed with serum hormone and ultrasound analysis. An hCG trigger (Ovidrel 250 µg, Merck) was administered on confirmation of one or two follicles at >17 mm and endometrial thickness >6 mm. Luteal support was achieved by progesterone pessaries (Oripro, Perrigo, Australia) 200 mg twice daily initiated on the day of embryo transfer.

### Programmed protocol

Oral oestradiol valerate (Progynova, Bayer, Australia) was taken in a step-up approach, from 2 mg daily up to 2 mg three times daily over a 12- to 14-day period. Once endometrial thickness was at least 6 mm on ultrasound assessment, progesterone pessaries 200 mg twice daily were commenced. Luteal phase support was provided by continuing the oestradiol and progesterone until the early pregnancy ultrasound at 7 weeks gestation.

### Frozen embryo transfer

Embryo transfer was performed 2–5 days after the LH peak/hCG trigger/initiation of vaginal progesterone, depending on the developmental stage at which the embryo had been frozen. Single embryo transfer was encouraged, and a maximum of two embryos could be transferred simultaneously. An embryo was determined to be of good quality if it was graded A or B by the Gardner system at the appropriate stage for its developmental day (Gardner & Schoolcraft 1999).

### Fresh cycle details

Women underwent controlled ovarian hyperstimulation (COH) using either a GnRH antagonist (Orgalutran, MSD, NJ, USA, or Cetrotide, Merck, Darmstadt, Germany) or agonist protocol (Synarel, Pfizer, West Ryde, Sydney, Australia, or Lucrin AbbVie Pty Ltd, Mascot, Sydney, Australia) together with FSH (Gonal-F, Merck or Puregon, MSD) at a dose selected based on the patient’s age, ovarian reserve and any previous response to COH. Follicular development was monitored by serum E2 levels and vaginal ultrasound, with FSH dose adjusted accordingly based on individual patient’s ovarian response. Final oocyte maturation was triggered by hCG injection (Ovidrel 250 µg) or GnRH agonist (Lucrin 200 µg) administered when three or more follicles measured ≥17 mm. Oocytes were retrieved transvaginally 36–38 h later. Cumulus–oocyte complexes were inseminated or microinjected approximately 39–40 h after the trigger injection. Fertilisation was assessed 16–18 h postinsemination/injection. Embryos were cultured using the G-series sequential medium (Vitrolife AB, Vasta Frolunda, Sweden). Embryos were assessed as described elsewhere (Alpha Scientists in Reproductive & Embryology 2011). Fertilised oocytes and cleavage stage embryos were frozen/thawed using the FreezeKit/ThawKit Cleave (Vitrolife AB), and blastocysts were vitrified/warmed using RapidVit/RapidWarm Blast (Vitrolife AB) in accordance with the manufacturer’s instructions. Thirty minutes prior to FET, the embryos were placed in EmbryoGlue (Vitrolife AB).

Statistical analysis was performed with the assistance of Christopher Backstrom (Statistiska Konsultgruppen). Baseline characteristics (Table 2) were regarded as independent observations. For comparisons between groups with non-ordered categorical variables, the chi-square test was used, and for continuous variables, the Kruskal–Wallis test was used. Univariable odds ratios (OR) for live birth and miscarriage rates relative to letrozole were calculated by a logistic regression model allowing for repeated measures with compound symmetry covariance structure. The ORs were also adjusted for by age at oocyte pick-up (OPU), embryo quality and stratified by natural cycle length.

**Table 2:** Clinical baseline characteristics and embryological data of women undergoing FET. For continuous variables, the mean (s.d.) is shown. For binary variables, the number (proportion) of women is shown. Statistical tests identified that there was a significant difference between the groups for all variables (*P <* 0.05).

Characteristics	Letrozole	Natural	FSH OI	Programmed
*n*	830	4565	382	283
Age at OPU (years)	32.6 (4.4)	34.2 (4.8)	31.8 (4.4)	35.4 (6.1)
Age at FET (years)	34.1 (4.3)	35.6 (4.8)	33.5 (4.5)	37.5 (6.3)
Oocyte or embryo recipient	16 (1.9)	53 (1.2)	3 (0.8)	116 (41.0)
Aetiology of infertility				
Male factor	237 (29.6)	1745 (38.8)	109 (29.4)	57 (20.6)
Endometriosis	113 (13.6)	925 (20.3)	67 (17.5)	51 (18.0)
Bilateral tubal occlusion	39 (5.7)	387 (9.8)	20 (6.1)	24 (9.5)
Idiopathic	140 (17.2)	1340 (29.8)	46 (12.2)	17 (6.0)
Natural cycle type				
Short (<28 days)	64 (8.1)	1156 (25.7)	15 (7.2)	15 (8.2)
28–35 days	276 (34.7)	2986 (66.4)	64 (30.6)	27 (14.7)
Long (>35 days)	455 (57.2)	356 (7.9)	130 (62.2)	142 (77.2)
Day of embryo transfer				
Cleavage (day 2 or 3)	77 (9.3)	658 (14.4)	38 (9.9)	108 (38.2)
Day 5	753 (90.7)	3907 (85.6)	344 (90.1)	175 (61.8)
Good quality embryo transferred	668 (80.5)	3629 (79.5)	322 (84.3)	206 (72.8)
Single embryo transfer	724 (87.2)	3883 (85.1)	312 (81.7)	221 (78.1)

## Results

Over the 5-year study period, there were 6072 FET cycles conducted. Of these, 6060 were included for analysis. Twelve cycles were excluded due to missing cycle or pregnancy outcome data.

The majority of women (4565, 75.3%) had undergone a natural cycle, while 830 (13.7%) had proceeded with a letrozole OI cycle. The baseline characteristics of women undergoing each cycle type are shown in Table 2. The women undergoing a programmed cycle had an oocyte collection at an older age (35.4 years) compared to other cycles (mean: 31.8–34.2 years). They were also more likely to be an oocyte or embryo recipient (41% vs 0.8–1.9%). Of the cycles for which data were available, women having a natural regimen were more likely to have a regular cycle, while women undergoing a letrozole OI regimen were more likely to have a longer cycle.

Cleavage stage embryo transfers were more common in women having a programmed cycle (38.2%) compared to the other cycles (9.3–14.4%). There was little difference however between the quality of embryos transferred with 72.8–84.3% of embryos transferred being graded as good quality. Two embryos were more likely to be transferred in a programmed cycle (21.9%) compared to other cycles (12.8–18.3%).

The pregnancy outcomes of the FET cycles are shown in Table 3. The LBR resulting from letrozole OI cycles was significantly higher compared to natural (OR: 1.27 (1.07–1.49)) or programmed cycles (OR: 2.36 (1.67–3.34)). There was no significant difference between the effect of letrozole compared to FSH OI cycles (OR: 0.99 (0.76–1.28) (Table 4). When adjusted for age and embryo quality, the significant differences were only maintained for letrozole OI above programmed cycles (OR: 1.92 (1.33–2.77)) (Table 4).

**Table 3:** Pregnancy outcomes of FET cycles. The cases counted were defined in line with the ESHRE consensus (Zegers-Hochschild *et al.* 2017). The table indicates the number (percentage rate) of each variable. The live birth delivery rate represents the number of deliveries that resulted in at least one live birth. Spontaneous miscarriage includes cycles which ended in a spontaneous miscarriage or missed miscarriage.

	Letrozole	Natural cycle	FSH OI	Programmed
Embryo transfers, *n*	830	4565	382	283
Live birth delivery^a^	266 (32.0)	1253 (27.4)*	124 (32.5)	47 (16.6)*
Clinical pregnancy^a^	336 (40.5)	1571 (34.4)	158 (41.4)	70 (24.7)
Biochemical pregnancy^b^	50 (13.0)	203 (11.4)	24 (13.2)	27 (27.6)
Spontaneous miscarriage^c^	59 (17.6)	280 (17.8)	28 (17.7)	22 (31.4)*
Ectopic pregnancy^b^	4 (1.0)	14 (0.8)	4 (2.2)	1 (1.0)
Stillbirth^d^	3 (1.1)	12 (0.9)	0 (0.00)	0 (0.00)
Neonatal death^e^	1 (0.4)	1 (0.1)	0 (0.00)	0 (0.00)
Multiple delivery (from SET)^e^	11 (4.6)	38 (3.4)	4 (4.0)	0 (0.00)
Multiple delivery (from DET)^e^	6 (22.2)	35 (24.3)	8 (32.0)	4 (36.4)

The denominator for the percentage rates calculated was ^a^per embryo transfer cycle, ^b^per pregnancy demonstrated by a positive hCG, ^c^per clinical pregnancy, ^d^per birth, ^e^per live birth delivery from single embryo transfer (SET) or double embryo transfer (DET). *Statistical significance between letrozole and the other regimens for live birth delivery, biochemical pregnancy and spontaneous miscarriage rate was assessed using the chi-square test with significance defined as *P <* 0.05.

**Table 4:** Pregnancy outcomes from FET cycles, compared to letrozole OI – univariable analysis and adjusted for age at the time of OPU and embryo quality. The odds ratio (95% CI) of the LBR and spontaneous miscarriage rate for each cycle regimen, compared to letrozole OI FETx cycles is shown.

	Effect of letrozole compared to		
	Natural	FSH OI	Programmed
LBR			
Univariable	**1.27 (1.07–1.49)**	0.99 (0.76–1.28)	**2.36 (1.67–3.34)**
Adjusted*	1.12 (0.95–1.34)	1.08 (0.81–1.42)	**1.92 (1.33–2.77)**
Spontaneous miscarriage			
Univariable	1.00 (0.73–1.37)	1.09 (0.62–1.89)	**0.46 (0.26–0.83)**
Adjusted*	1.13 (0.81–1.56)	1.09 (0.61–1.94)	0.58 (0.31–1.07)

*Adjusted for age at time of OPU and embryo quality.

Significantly different results are given in bold.

The LBR was also examined according to the woman’s recorded cycle length (Table 5). A significantly higher LBR was identified in women with a normal cycle length following letrozole OI compared to natural (OR: 1.44 (1.10–1.89)) or programmed (OR: 4.56 (1.45–14.34)) cycles. There was no significant difference in LBR between letrozole OI and the other regimens for women with a short cycle less than 28 days. For those women with a cycle longer than 35 days, letrozole OI was only beneficial when compared to programmed cycles (OR: 2.02 (1.24–3.29)).

**Table 5:** Odds ratio for LBR from FET cycles stratified by cycle length, compared to letrozole OI. The table indicates the odds ratio (95% CI) for LBR for each FET cycle type, stratified by cycle length, compared to letrozole OI FETx cycles.

	Effect of letrozole, compared to		
	Natural	FSH OI	Programmed
Short (<28 days)	0.95 (0.52–1.74)	0.85 (0.26–2.85)	2.10 (0.43–10.38)
28–35 days	**1.44 (1.10–1.89)**	1.69 (0.89–3.21)	**4.56 (1.45–14.35)**
Long (>35 days)	1.09 (0.80–1.47)	0.83 (0.56–1.24)	**2.29 (1.41–3.71)**

Significantly different results are given in bold.

The rate of spontaneous miscarriage only differed from letrozole OI cycles when programmed cycles were used (OR: 0.46 (0.26–0.83)) (Table 4). This effect however was not maintained when adjusted for age and embryo quality (OR: 0.58 (0.31–1.07)) (Table 4).

The duration of the follicular phase of each cycle type was assessed (Table 6). For those letrozole OI, natural and FSH OI cycles that led to a live birth, the mean duration of the follicular phase was 15–16 days. Programmed cycles had a slightly longer follicular phase of 18 days. FSH OI cycles could adequately prepare the endometrium following only 5 days of stimulation. Other cycle types however needed 8–10 days of a follicular phase if the cycle was to lead to a live birth. Embryo transfers which were performed after a follicular phase of 30–38 days did not result in a live birth (Table 6).

**Table 6:** Secondary outcomes from FET cycles. The follicular phase was defined as the number of days from a woman’s first day of her last menstrual period to her natural LH surge (for letrozole OI, natural cycles), trigger (FSH OI cycles) or commencement of progesterone (programmed cycles).

	Letrozole	Natural cycle	FSH OI	Programmed
Length of follicular phase to achieve positive bHCG (days)				
Minimum	9	8	5	9
Length of follicular phase to achieve live birth (days)				
Minimum	10	8	5	9
5th percentile	11	11	8	10.4
Mean	15.47	15.02	16.07	18.17
95th percentile	22	21	26	26.6
Maximum	30	34	38	28
Blood or urine samples taken during cycle (mean)	2.6	2.1	5.5	1.0

Statistical tests identified that there was a significant difference (*P <* 0.05) between the groups for all parameters.

Compared to letrozole OI and natural cycles, the number of blood or urine tests required by women undergoing an FSH OI cycle was at least two times greater (Table 6).

## Discussion

The current study suggests that using letrozole to achieve endometrial preparation prior to FET may yield a higher LBR, compared to natural and programmed cycles (Tables 3 and 4). This however should be interpreted with caution as it may not apply for all women. Further, the outcome data from this retrospective cohort may be confounded by variables which could not be accounted for. The women being chosen for each cycle type are likely to represent groups with variable prognoses. In total, 57.2% of women having a letrozole OI cycle and 62.2% having an FSH OI cycle had long cycles (Table 2). This is in contrast to only 7.9% of women chosen to have a natural cycle. The most common reason for oligomenorrhoea is polycystic ovarian syndrome (PCOS). It is likely that women with PCOS are over-represented in the OI cycles, and as a result, they may have a better prognosis due to their higher ovarian reserve. However, in programmed cycles, where 77.2% of women were defined as having a long cycle, this may actually represent those women with ovarian insufficiency and hence a poor prognosis. This may be partially counterbalanced by the fact that 41% of those women were a recipient of donor oocytes or embryos.

The type of regimen to use for women with a normal length cycle is frequently debated. It is proposed that the use of ovulation induction with either letrozole or FSH, even in the presence of spontaneous folliculogenesis and an LH surge, may enhance endometrial preparation. The current study suggests that letrozole OI improved the LBR for women with a regular cycle compared to a natural cycle (OR: 1.44 (1.10–1.89)) (Table 5). It may be that while women seem to demonstrate a normo-ovulatory cycle, the quality of their endometrial preparation, driven by folliculogenesis and corpus luteum formation, may be impaired in a way that we currently lack a diagnostic tool for. The use of letrozole OI may circumvent this defect.

For women with a long cycle, programmed or OI regimens are more commonly employed. The current study demonstrated that letrozole yielded a higher LBR, compared to programmed cycles (OR 2.02 (1.24–3.29)) in this population group (Table 5). In a study of women with PCOS, Zhang *et al.* demonstrated that after adjusting for age, letrozole OI led to a higher LBR (Zhang *et al.* 2019). In one of the few RCTs involving letrozole OI for FET, Li *et al.* demonstrated that in women with ovulatory disorders, a letrozole endometrial preparation regimen led to a higher LBR compared to a programmed cycle (Li *et al.* 2014).

The inferiority of programmed cycles quite consistently across this current study and previously published literature may be linked to the importance of the corpus luteum in establishing and maintaining a pregnancy. It has recently been highlighted that pregnancies of women who have conceived following FET have demonstrated a higher incidence of hypertensive disorders of pregnancy (Ishihara *et al.* 2014, Sha *et al.* 2018). This is thought to be associated with the absence of the corpus luteum and its secretion of hormones such as relaxin and the subsequent placentation events which occur (Ginstrom Ernstad *et al.* 2019, von Versen-Hoynck *et al.* 2019, Dall’Agnol & Garcia Velasco 2020). The pregnancy outcomes demonstrated in the current study further support the important role the corpus luteum plays in the establishment of a pregnancy.

The strength of this study is that, to our knowledge, it is the only study published which includes a comparison of the four most commonly used endometrial preparation techniques within one cohort (Table 1). The sample size is extensive compared to previously published literature hence potentially ensuring that the sample of women studied more closely approximates the population of women undergoing FETs.

This study is also unique in that it explored the duration of the follicular phase of a cycle required to lead to a pregnancy resulting in a live birth. This can serve as a guide when assessing hormonal results suggestive of either short or long follicular phase lengths. It suggests, for example, in women having a letrozole OI FET cycle, if a peak oestrogen is reached prior to day 10, it may be worthwhile cancelling the cycle. The absence of live births prior to this may indicate that there is an inadequate length of time available for appropriate oestrogen levels to act on the endometrium.

At the other end of the spectrum, 95% of letrozole OI FET live births were achieved if the follicular phase was up to 22 days in duration, while the longest follicular phase at which a live birth was achieved was 30 days. Previous studies exploring the endocrine profiles of ovulatory women taking letrozole have indicated a narrow CI for the follicular phases observed (13.1 ± 0.3 days) (Jirge & Patil 2010). The current paper highlights that many women will not fall into this narrow definition of a follicular phase length and so it is worthwhile persisting with the cycle, even in the face of an apparently ‘long’ follicular phase. In fact, of the 31 women who had a follicular length of 22–30 days in duration with a letrozole OI cycle, 12 achieved a live birth following embryo transfer, giving an LBR of 39%.

The current study is however limited by its retrospective cohort, non-randomised design. Details of the woman’s baseline fertility prognosis and data regarding previous cycles, including fresh cycle details, were not assessed. This makes it challenging to assess if the findings are specific to a particular group of women defined by certain characteristics or if they have been confounded by other factors such as duration or aetiology of infertility or BMI.

In the absence of clear evidence which definitively demonstrates an improved LBR with one particular regimen, other elements of a FET cycle should be considered. These include the cost to the patient or the healthcare system in terms of resources required to track a cycle (time, equipment and personnel) or medication-related costs. Around twice as many blood or urine tests were required to manage an FSH OI cycle compared to a natural or letrozole OI cycle (Table 6). Some patients may be particularly averse to using injectable medications and so an oral letrozole regimen may be more suitable. Further, when assessing the burden of treatment, the cost of FSH preparations needs to be considered, which could be up to 20 times that of letrozole.

Noting their different mechanisms of action, it is likely that each endometrial preparation regimen may better suit women defined by particular characteristics. In order to determine this, a prospective study randomising women stratified by their baseline fertility prognosis and diagnosis would be worthwhile. Due to the large number of women required however to achieve sufficient power, an alternative is to continue performing retrospective studies. It is critical however that clinics focus on the accurate collection and regular auditing of uniform data to ensure appropriate big data analyses can be performed.

Any future studies should also examine the long-term pregnancy and neonatal outcomes as the cycle type may influence the occurrence of hypertensive disorders of pregnancy or growth restriction (Dall’Agnol & Garcia Velasco 2020). The differences in pregnancy outcomes demonstrated in this study also highlight the importance of clearly stating the type of FET cycle performed, when comparing ‘fresh’ to ‘frozen' embryo transfers. It is becoming more clear that not all FET cycles are equivalent.

## Conclusion

This retrospective cohort study demonstrated a significantly higher LBR and lower miscarriage rate in women having FET in letrozole OI cycles compared to programmed cycles. The difference in LBR between letrozole OI and FSH OI cycles was not found to be significant, while that between letrozole OI and natural cycles was no longer significant after adjusting for potential confounders. Patients having an FSH OI cycle incurred a higher expense for drugs and had twice as many test occasions compared to letrozole OI patients. This indicates that when considering the cost and burden of treatment, the preferred OI endometrial preparation for FET is letrozole.

## Declaration of interest

The authors declare that there is no conflict of interest that could be perceived as prejudicing the impartiality of the research reported.

## Funding

This work did not receive any specific grant from any funding agency in the public, commercial, or not-for-profit sector.

## Author contribution statement

M A and C S conceived the study question and designed the type of data to be extracted from the database. J C extracted the data from the clinical database. M A performed the literature review, was responsible for ethics approval application and collated the data for review by the statistician (C B acknowledged below). M A wrote the paper, while C S and H S provided editorial support to draw final conclusions and prepare the article for publication.
